# Effect of screening and brief intervention for illegal drug use in Southern California

**DOI:** 10.1186/1940-0640-7-S1-A10

**Published:** 2012-10-09

**Authors:** John Clapp, Susan I Woodruff

**Affiliations:** 1 San Diego State University Center for Alcohol & Drug Studies, San Diego, CA, USA

## 

The California Screening, Brief Intervention, and Referral to Treatment (CASBIRT) program provided services to over 120,000 emergency/trauma patients throughout ethnically diverse San Diego County from 2007–2010. This study examined outcomes for drug users screened in emergency departments (ED) throughout the county. Universal screening using the ASSIST was offered to all capable adult patients by trained bilingual/bicultural health educators in 12 ED/trauma centers over a three-year period. Patients who screened positive on the Alcohol, Smoking, and Substance Involvement Screening Test (ASSIST) were given brief motivational feedback/intervention matched to risk level. A random sample of patients was targeted for a follow-up telephone interview six months later. This analysis included participants who reported risky drug use in addition to risky alcohol use at their initial screening (N = 1171). About 32% of the sample (n = 373) were actually interviewed by telephone at follow-up. We conducted analyses per intent to treat and recoded missing follow-up values using the “last value carried forward” approach. Half of the resulting sample of 1171 patients was male; the average age was 37 (SD = 13.3). The sample was 44% non-Latino white, 35% Latino, 15% black, and 7% other racial/ethnic groups. The substance most commonly reported was marijuana (29%) followed by methamphetamine (13%) and heroin (7%). There was a significant entry-to-follow-up reduction in the percent reporting any use of illegal drugs in the past month (53% to 38%, F = 35.33, p < 0.001). Days of use of illegal drugs also showed reductions, from a mean of five days to four days (F = 63.74, p < 0.001). These results, based on a conservative analysis approach, demonstrated that CASBIRT had a statistically significant positive effect on self-reported illegal drug use.

